# Editorial: Phytotherapeutic alternatives in veterinary medicine, vol II

**DOI:** 10.3389/fvets.2023.1174494

**Published:** 2023-03-29

**Authors:** Nora Mestorino, Lloyd Reeve-Johnson

**Affiliations:** ^1^Laboratorio de Estudios Farmacológicos y Toxicológicos -LEFyT-, Facultad de Ciencias Veterinarias, Universidad Nacional de La Plata, La Plata, Argentina; ^2^One Health, Faculty of Science, Health, Education and Engineering, University of Sunshine Coast, Queensland, QLD, Australia; ^3^Goyd Project Solutions, Queensland, QLD, Australia

**Keywords:** Modified Gegen Qinlian Decoction (MGQD), coccidiosis, Alfalfa saponins, growth performance, *Macleaya cordata* (Willd.) R. Br., anti-inflammatory activity, *Aeromonas hydrophila*, polydatin

Intensive animal husbandry systems are often associated with higher population densities and extensive use of artificial feeds. Outbreaks of bacterial and parasitic diseases are a major limiting factor for animal farming and producers often use large amounts of antimicrobials, anthelmintics, disinfectants, and pesticides to control mortality and avoid economic losses. Due to adverse effects on the environment, and on animal and human health, the usefulness of these practices has been questioned. Using plant-based therapies within animal production has shown potential as a natural and biodegradable source of Compounds with activity against a variety of pathogens. Plant derived therapy plays an important role ensuring global health, especially in developing countries. The use of phytotherapy has a long history for prevention, clinical treatment, and cure of diseases and well as growth and health promoters in animal nutrition. Medicinal herbs, derivatives, and organic products may be considered one of the most important holistic alternatives to treat livestock globally.

Phytotherapy has been shown to have a wide variety of applications in humans, including, cholesterol control; supportive treatment with some cancers; anti-inflammatory activity (1, 2); antimicrobial and antiparasitic activity, and is becoming an essential source for modern drug development targets (3).

The antimicrobial and antiparasitic activity of many plant extracts deserves particular interest for the potential to mitigate resistance problems with other types of drugs. In veterinary medicine, phytotherapy is a promising alternative providing benefits to growth and rate of live weight gain in food animal production, avoiding the use of antimicrobials and reducing the risk of selecting for antimicrobial resistance (4). Multiple mechanisms of resistance by microorganisms have caused a very concerning decline in therapeutic efficacy of antibiotics over time making it necessary to seek new strategies for control.

This Research Topic has collated papers to improve our understanding of the activity, safety, and efficacy, of botanical medicines. In this Special Topic, there are four papers covering these aspects.

In the study by Peng et al. the protective effect of the active ingredient Modified Gegen Qinlian Decoction (MGQD) for the treatment of chicken coccidiosis was explored by detection through network pharmacology and RNA-Seq technology of differentially expressed genes (DEGs). Gegen Qinlian Decoction is a long-standing Chinese herbal compound for diarrhea and dysentery treatment. The authors were able to predict the therapeutic mechanism of the active ingredient MGQD for chicken coccidiosis by pathway enrichment analysis of intersecting targets. Peng et al. indicate that the mechanism of action of MGQD in the treatment of chicken coccidiosis is related to its anti-inflammatory and antioxidant properties and inhibition of oncogene transcription and action. Targets were related to SRD, STAT3, and PPARG. This is the firs study to explore the efficacy and mechanism of the effective active ingredient of MGQD against coccidiosis of chickens, providing support for future drug development.

In the study presented by Yang et al. the potential effects of alfalfa saponins on production performance, serum biochemical factors, and immune factors in sheep were determined. The results obtained by the authors confirm the effects of alfalfa saponins on growth performance and meat quality in Small-Tailed Han sheep, with the most efficient supplementation at a ratio dose of 10 g/kg. Alfalfa saponins also had effects on serum levels of biochemical factors, which were both dose and time-dependent. Supplying alfalfa saponins increased the serum concentrations of IgA, IgG, IgE, IgM, IL-1, IFN-a and IFN-b. The concentration of alfalfa saponins of 10 g/kg during 14 consecutive days was suggested as the optimal ratio for sheep health.

*Macleaya cordata* (Willd.) R. Br. is a Chinese medicinal plant, of the poppy family, commonly used externally to treat inflammatory-related diseases (arthritis, wounds, swelling, and carbuncles). Alkaloids from *M. cordata* exhibit anti-inflammatory activity, but the anti-inflammatory activity and mechanism of action of protopine alkaloids remain unknown. Dong, Z., et al. evaluated the anti-inflammatory activity of protopine total alkaloid (MPTAs) in *Macleaya cordata* (Willd.) R. Br., using animal models of acute inflammation (rat paw edema, and mouse ear swelling). Protopine, and allocryptopine, the two main active components of MPTA, were identified. Potential targets and signaling pathways of MPTA's anti-inflammatory effects were determined by means of tools such as Swiss Target Prediction, GeneCards, and STRING combined with molecular docking results. Using animal models of inflammation, the authors demonstrated the anti-inflammatory activity of MPTA, and through network pharmacology and molecular docking results obtained, suggest that MPTA may exert its anti-inflammatory effects by acting on targets such as MTOR, SRC, MAPK3, PIK3CA, and PTGS2.

Finally, the article of Dong, J., et al. addresses a topic of critical environmental importance specifically the use of natural compound in aquaculture. This activity has developed rapidly in recent years due to the growing demand for aquatic products. As in other animal production, diseases caused by bacterial pathogens generate a serious economic loss. The introduction of antibiotics in aquaculture reduced mortality from infectious diseases, but nevertheless, the appearance and spread of antimicrobial resistance (AMR) has caused treatment failure and also the presence of residues with the consequent environmental contamination. So, it is urgent to evaluate new strategies to combat infections caused by resistant bacterial strains. Dong, J., et al. identified aerolysin as a potential target to develop drugs from natural compounds against *Aeromonas hydrophila* infections. *A. hydrophila*, a Gram-negative bacterium widely distributed in aquatic environments, is responsible for various diseases that affect farmed fish. The authors found, through Western blot and qPCR assays, that polydatin (which has no inhibitory effect against the growth of *A. hydrophila*) could decrease the haemolysis caused by aerolysin because adding polydatin decreased the production of aerolysin by regulating the gene encoding aerolysin is lowered. It was also determined through animal and cell feasibility studies that polydatin could reduce the pathogenesis of *A. hydrophila*. Thus, Dong, J., et al. provided a novel approach and a candidate for the treatment of resistant *A. hydrophila* infections in aquaculture.

In summary, the results of the above-mentioned studies corroborate that research on plants for medicinal purposes is essential to facilitate the emergence of options to obtain pharmacological alternatives for human and animal health, within the “One Health” concept of holistic approaches to the interdependent health of man, animals and the environment.

## Author contributions

Both authors listed have made a substantial, direct, and intellectual contribution to the work and approved it for publication.
